# Anti-Inflammatory Preconditioning by Agonists of Adenosine A1 Receptor

**DOI:** 10.1371/journal.pone.0002107

**Published:** 2008-05-07

**Authors:** Sigal Nakav, Cidio Chaimovitz, Yuval Sufaro, Eli C. Lewis, Gad Shaked, David Czeiger, Moshe Zlotnik, Amos Douvdevani

**Affiliations:** 1 Department of Clinical Biochemistry, Soroka Medical University Center and Ben-Gurion University of the Negev, Beer-Sheva, Israel; 2 Department of Nephrology, Soroka Medical University Center and Ben-Gurion University of the Negev, Beer-Sheva, Israel; 3 Department of General Surgery, Soroka Medical University Center and Ben-Gurion University of the Negev, Beer-Sheva, Israel; Instituto Oswaldo Cruz and FIOCRUZ, Brazil

## Abstract

**Background:**

Adenosine levels rise during inflammation and modulate inflammatory responses by engaging with four different G protein-coupled receptors. It is suggested that adenosine exhibits pro-inflammatory effects through its A_1_ receptor (A_1_R), and anti-inflammatory effects through A_2A_ receptor (A_2A_R). Therefore, understanding of the mechanisms that govern adenosine receptor regulation may advance treatment of various inflammatory disorders. We previously reported that peak A_1_R expression during leukocyte recruitment, is followed by a peak in A_2A_R during inflammation resolution.

**Principal Findings:**

Here, we examined whether A_1_R activation sequentially induces A_2A_R expression and by this reverses inflammation. The effect of adenosine on A_1_R mediated A_2A_R expression was examined in peritoneal macrophages (PMΦ) and primary peritoneal mesothelial cells (PMC) *in vitro*. Induction of A_2A_R was inhibited by pertussis toxin (PTX) and partly dependent on A_2A_R stimulation. Administration of A_1_R agonists to healthy mice reduced A_1_R expression and induced A_2A_R production in PMC. Mice that were preconditioned with A_1_R agonists 24 hours before *E. coli* inoculation exhibited decreased TNFα and IL-6 sera levels and reduced leukocytes recruitment. Preconditioning was blocked by pretreatment with A_1_R antagonist, as well as, or by late treatment with A_2A_R antagonist, and was absent in A_2A_R^−/−^ mice.

**Conclusions:**

Our data suggest that preconditioning by an A_1_R-agonist promotes the resolution of inflammation by inducing the production of A_2A_R. Future implications may include early treatment during inflammatory disorders or pretreatment before anticipated high risk inflammatory events, such as invasive surgery and organ transplantation.

## Introduction

Over the past few years, a vast number of investigations have reported the involvement of adenosine in the anti-inflammatory process [1], [2]. Adenosine is an endogenous purine nucleoside that is constitutively present in the extracellular spaces at low concentrations. However, in metabolically-stressful conditions such as tissue damage, ischemia and inflammation, adenosine dramatically increases its extracellular levels. Extracellular adenosine levels have been observed to increase by dephosphorylation of ATP in non-immune and immune cells [1] and then to be released through the action of specialized nucleoside transporters [3]. Extracellular adenosine interacts with at least four different receptor subtypes [4]–[6]. The A_2A_ receptor (A_2A_R) interacts with the G protein G_s_ and the A_2B_ receptor (A_2B_R) interacts with the G proteins G_s_ and G_q_ to induce adenylyl cyclase activity and elevate cAMP levels. In contrast, ligation of adenosine to the A_1_ receptor (A_1_R) or to the A_3_ receptor (A_3_R), through interaction with members of the G_i_/G_o_ family, inhibits adenylyl cyclase activity and decreases cAMP levels [7]. A_1_R exerts a pro-inflammatory response by enhancing phagocytosis [8], promoting chemotaxis [9], [10] and enhancing neutrophils adherence to endothelium during inflammatory process [11]. In contrast, engagement of A_2A_R inhibits neutrophils adherence to endothelium during inflammation [12] and inhibits the activation of neutrophils, monocytes platelets and T-cells [13]–[15]. In animal models, A_2A_R-agonists can prevent lethal response to bacterial LPS and sepsis [16], [17].

Since each of these receptor subtypes has a unique physiological profile and a particular affinity to its ligand, the inflammatory state is determined by both extracellular adenosine concentrations and by the distribution and expression levels of its receptor subtypes. It has been shown that the expression of adenosine receptors is regulated by factors that are involved in the inflammatory response, such as LPS [18], pro-inflammatory cytokines [19]–[21], growth factors [22], [23] and glucocorticoids [24]. Recently, we have shown in a model of peritonitis that shortly following inoculation, A_1_R mRNA and protein levels are upregulated on peritoneal mesothelial cells (PMC), reaching a peak in the initial phase of the inflammatory process [19]. Interestingly, concomitant with the resolution phase of peritonitis, we observed a decrease in A_1_R expression levels and an elevation of adenosine and A_2A_R levels. The coordinated kinetics of adenosine and its receptors led to the hypothesis that adenosine differentially regulates its own receptors. Since the two receptors, A_1_R and A_2A_R, have opposing biological effects, and A_1_R domination precedes the elevation of A_2A_R, we sought to examine whether A_1_R activation would be one of the factors that trigger the anti-inflammatory phase, and whether this action is mediated by upregulation of the A_2A_R.

To test our hypothesis, we examined the effect of adenosine receptor agonists and antagonists *in vivo* in a model of peritonitis induced by *E. coli* inoculation. This model has particular clinical significance because peritonitis is commonly caused by pathological processes of the gastrointestinal tract or as a complication of abdominal surgery. *In vitro*, we examined the regulation of the receptors on the cell surface of PMΦ, which are the first line of cellular defense against bacterial invasion in the peritoneum [25], and on PMC, the cells that line the peritoneal membrane and therefore play an important role in transferring inflammatory signals from the peritoneal cavity to the blood vessels [26]–[30]. We demonstrate that A_1_R activation triggers the switching of adenosine receptor subtype from A_1_R to A_2A_R. By the anti-inflammatory effects of the ligation of adenosine to the A_2A_R, the described receptor subtype switch alters the progression of inflammation toward resolution.

## Materials and Methods

### Mice, bacterial strains and drugs

CD1 female mice aged 10 to 12 weeks (Harlan, Jerusalem, Israel) were maintained in the animal laboratory of the Soroka Medical Center. Experiments were conducted with the permission of the Israel Committee for Animal Experiments. A_2A_R^−/−^ mice whose phenotype is well established in the literature were graciously kindly donated by Catherine Ledent (Université Libre de Bruxelles) [31].

*Escherichia coli (E. coli)* were grown in Luria-Bertani broth (Conda Laboratories, Madrid, Spain) and harvested during the log phase. Bacteria aliquots in Luria-Bertani broth containing 30% glycerol were stored frozen at −70°C. Adenosine (Adenocor) was purchased from Sanofi Winthrop (Auckland, NZ). A_2A_R antagonist 4-(2-[7-Amino-2-(2-furyl)[1], [2], [4]triazin-5-ylamino]ethyl) phenol (ZM241385) was purchased from Tocris Cookson (Ellisville, MS). Pertussis toxin (PTX) and other Adenosine receptor agonists and antagonists were purchased from Sigma (Rehovot, Israel): A_1_R agonists N^6^-cyclohexyadenosine (CHA) and 2-Chloro-N^6^-cyclopentyladenosine (CCPA); A_1_R antagonist 8-cyclopentyl-1, 3-dipropylxanthine (DPCPX); A_2A_R agonist 2-*p*-(carboxyethyl) phenethylamino-5′-N-ethylcarboxamideadenosine hydrochloride (CGS21680); A_3_R antagonist 9-Chloro-2-(2-furanyl)-5-((phenylacetyl)amino)-[1], [2], [4]triazolo[1,5-c] quinazoline (MRS1220); A_2B_R antagonist 8-[4-[((4-Cyanophenyl)carbamoylmethyl)oxy]phenyl]-1,3-di(n-propyl)xanthine hydrate (MRS1754).

### Induction of peritonitis and treatment protocol

Peritonitis was induced in mice by intraperitoneal (i.p.) inoculation of a sub-lethal dose of *E. coli* (3.6×10^9^ CFU). Adenosine agonists and antagonists were injected i.p. before *E. coli* inoculation.

### Sera and peritoneal lavage fluids collection, leukocyte counting and cytokine detection

At different time points after *E. coli* inoculation, animals were anesthetized. 1 ml syringe flushed with heparin was used to draw intracardial blood sample. The samples were stored on ice before centrifugation at 1,000 g at 4°C for 10 minutes. The cell-free supernatants were collected and frozen at −20°C until assayed by ELISA. Peritoneal lavage was performed with 5 ml phosphate buffer saline (PBS) containing 2% BSA and 5 mM EDTA. After centrifugation at 400 g for 10 minutes, the cell-free supernatants were removed and frozen at −20°C until analysis. TNFα and IL-6 levels were determined by commercial ELISA kits (Biolegend, San Diego, CA and R&D Systems, Minneapolis, MN, respectively). Cells were washed once, and total leukocytes were counted after trypan blue staining using an improved Neubaur hemocytometer. Cell counts and ELISA were performed blindly on coded samples.

### Scraping of mice PMC

Following treatment, animals were anesthetized and PMC were scraped from the peritoneal membrane. The cells were stored on ice before centrifugation at 400g and 4°C for 10 minutes. Cells were harvested with lysis buffer for analyzing mRNA levels or with RIPA (150 mM NaCl, 50 mM Tris HCl pH-7.4, 1% NP-40, 0.25% Na deoxycholate, 1 mM EGTA) including protease inhibitor cocktail (Sigma) for analyzing protein levels.

### Preparation of cultured PMC and PMΦ

To prepare PMC, the peritoneum was removed from eight newborn (two-week old) mice and isolated, as previously described [32]. To assess the purity of mesothelial cells, samples of each PMC preparation were morphologically inspected, as previously described [33]. Cells were grown in M199 and supplemented with 10% heat-inactivated FCS, 2 mmol/l L-glutamine and 100 U/ml penicillin and 100 µg/ml streptomycin (Biological Industries, Bet Haemek, Israel). Experiments were performed on cells from the second to fourth passages. To prepare PMΦ, mice were injected intraperitoneally with 3 ml of 3% thioglycollate (Difco, Sparks, MD). After 3 days, peritoneal cells were collected by lavage and seeded onto 12-well plates in RPMI supplemented with 10% heat-inactivated FCS, 2 mmol/l L-glutamine and 100 U/ml penicillin and 100 µg/ml streptomycin. Non-adherent cells were subsequently removed by washing with PBS. In experiments, to simulate the graduate increase in adenosine levels found *in vivo*, cells were treated with increasing doses of adenosine or CHA with or without DPCPX (9 hours with 0.1 µM or 3 hours with 0.1 µM and then 6 hours with 1 µM or 3 hours with 0.1 µM, then 3 hours with1 µM and then 3 hours with 10 µM).

### mRNA analysis

Total RNA was extracted from PMC or PMΦ using the Versagene RNA cell kit (Gentra systems, Minneapolis, MN). cDNA was prepared as previously described [29]. Quantitative real time PCR (QPCR) assays were carried out for β-actin, GAPDH, A_1_R, A_2A_R, macrophage inflammatory protein-2 (MIP-2) and monocyte chemotactic protein-1 (MCP-1) with the following primers: β-actin sense: ′5-GGG TCA GGA GGA TTC CTA TG-′3, β-actin antisense: ′5-GGT CTC AAA CAT GAT CTG GG-′3, GAPDH sense: ′5-CAA TGC ATC CTG CAC CAC CAA-′3, GAPDH antisense: ′5-GTC ATT GAG AGC AAT GCC AGC-′3, A_1_R sense: ′5-TAC ATC TCG GCC TTC CAG GTC G-′3, A_1_R anti sense: ′5-AAG GAT GGC CAG TGG GAT GAC CAG-′3, A_2A_R sense: ′5-ATT TGT GCC AGC CAG GAA GCC-′3, A_2A_R antisense: ′5-GCA TCC GGG ACT TTA AAC CAC AGA-′3, MIP-2 sense: ′5-CTC CTC AGT GCT GCA CTG GT-′3, MIP-2 antisense: ′5-TCC CGG GTG CTG TTT GTT T-′3, MCP-1 sense: ′5-CTC ACC TGC TGC TAC TCA TTC-′3, MCP-1 anti sense: ′5-GCT TGA GGT GGT TGT GGA AAA-′3. cDNAs were diluted ×9, mixed with primers (0.2 mM) and Thermo start master mix (ABgene, Surrey, UK). Reaction was carried out in Rotor-Gene real time PCR machine (Corbett-Research, Northlake, Australia).

### Western blotting analysis

Cell lysates was centrifuged at 13,000 g for 30 minutes and then supernatants were collected for total protein determination by the BCA protein assay kit (Pierce, Rockford, IL). 30 µg of total protein from each sample was subjected to 10% SDS-PAGE under reducing conditions and after heating. The gels were blotted onto a PVDF membrane (Bio-Rad, Hercules, CA) and probed with the following specific antibodies: rabbit anti-adenosine A_2A_R (Santa Cruz Biotechnology, Santa Cruz, CA) or rabbit anti-A_1_R (Alpha Diagnostic International, San Antonio, TX) or goat anti-β-actin (Santa Cruz Biotechnology). The membrane was then probed with goat anti-rabbit immunoglobulins Ig-conjugated to peroxidase agent (Santa Cruz Biotechnology) or with donkey anti-goat IgG conjugated to peroxidase agent (Jackson Immuno Research laboratories, West Grove, PA). Antigen-antibody complexes were subsequently visualized by the EZ-ECL Chemiluminescence Detection kit for HRP (Biological Industries).

### Statistical Analysis

Data are presented as mean±SEM. Statistical analysis was performed by t-test or ANOVA followed by Tukey post test. P values below 0.05 were considered significant.

## Results

### Adenosine receptors exhibit unique expression kinetics in peritoneal leukocytes following bacterial inoculation

**Figure 1:**
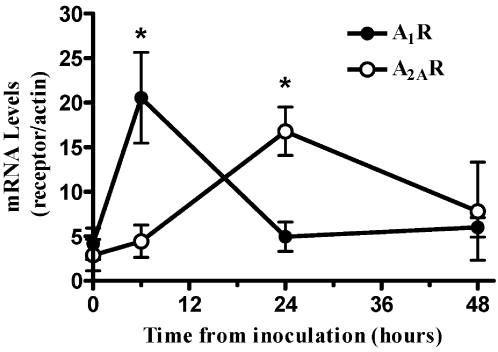
A_1_R and A_2A_R expression in peritoneal leukocytes during inflammation *in vivo.* Peritonitis was induced in mice by *E. coli* inoculation at a sub-lethal dose. To examine the dynamic expression of the two high-affinity adenosine receptors, A_1_R and A_2A_R, peritoneal lavage was performed at indicated time points. A_1_R and A_2A_R mRNA levels in peritoneal leukocytes were analyzed by real time PCR and normalized to β-actin levels. Data represent three experiments and are expressed as mean±SEM. * *p*<0.05, between expression levels of each receptor to expression at time 0, *n* = 5 for each experiment.

It has been shown that adenosine is upregulated during peritonitis [19]. We therefore examined the regulation of adenosine receptors in peritoneal leukocytes and found that the A_1_R and A_2A_R are upregulated during the first 48 hours of peritonitis. However each of the subtypes exerted unique kinetics. As shown in figure 1, A_1_R mRNA levels were maximal at 6 hours after inoculation and returned to basal levels at 24 hours, while A_2A_R mRNA levels gradually increased and reached maximum at 24 hours.

### Adenosine induces the expression of A_2_R in a dose-dependent manner

**Figure 2:**
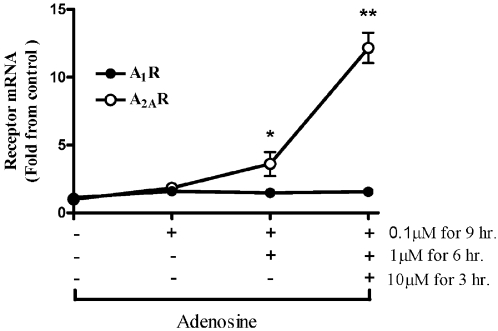
Effect of adenosine on A_2A_R and A_1_R levels *in vitro*. To simulate the gradual increase of adenosine that occurs during peritonitis, cultured primary PMC were treated with multiple and increasing concentrations of adenosine (0.1, 1 and 10 µM at 3 hour intervals). Total RNA was extracted after 9 hours and analyzed for A_1_R and A_2A_R mRNA levels. Results are normalized to β-actin. Data represent five experiments and are expressed as mean±SEM fold of control. * *p*<0.05, ** *p*<0.01 between expression levels of each receptor to expression at time 0, *n* = 3 for each experiment.

Since both adenosine and adenosine receptors are upregulated upon bacterial inoculation [19], we wanted to elucidate whether the regulation of adenosine receptors is adenosine-dependent. In order to simulate the gradual and accumulative increase of adenosine that is observed *in vivo*, we treated cultured PMCs with multiple and increasing concentrations of adenosine (0.1, 1 and 10 µM at 3 hours intervals). As shown in Figure 2, adenosine induced the expression of A_2A_R mRNA levels in a dose dependent manner. However, there was no change in A_1_R mRNA levels upon treatment with the different concentrations of adenosine.

### Adenosine regulates A_2A_R expression through A_1_R

**Figure 3:**
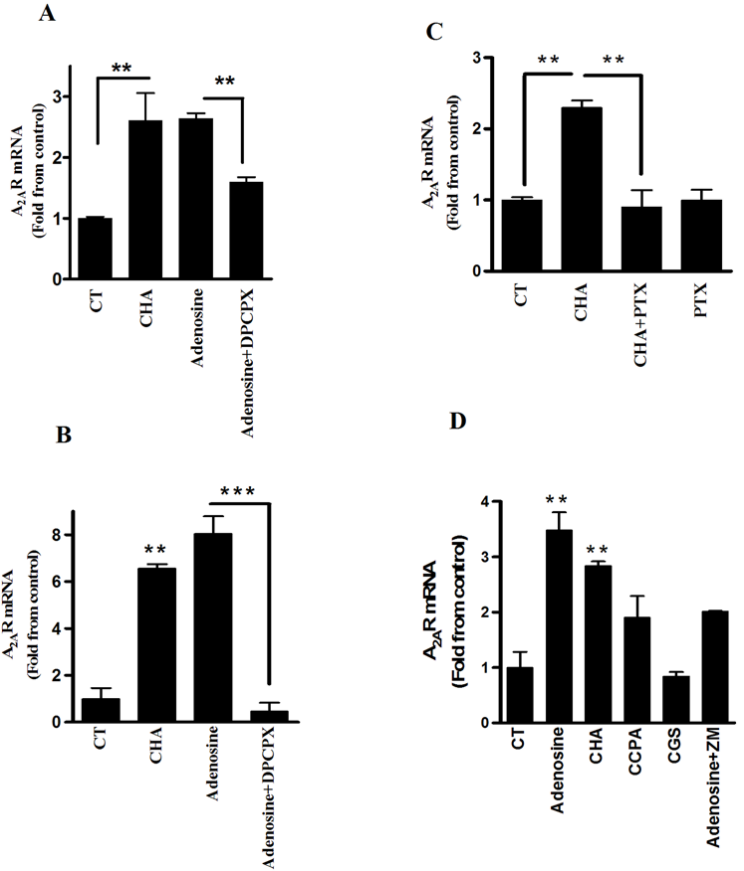
A_1_R trigger the induction of A_2A_R *in vitro*. (A) PMΦ or (B) PMC were exposed to increasing concentrations of adenosine or A_1_R agonist (CHA), (0.1, 1 and 10 µM 3 hours intervals) in the presence or absence of A_1_R antagonist (DPCPX, 50 nM, 30 min before treatment) (C) PMC were treated with PTX for 18 hr and then with increasing concentrations of CHA. (D) PMC were treated with increasing concentrations of adenosine, A_1_R agonists (CHA and CCPA) or A_2A_R agonist (CGS21680) in the presence or absence of A_2A_R antagonist (ZM241385, 50 nM). Total RNA was extracted from cells and analyzed for A_2A_R mRNA levels and normalized to β-actin. CT, non-treated cells. Data represent four experiments and are expressed as mean±SEM fold of control. ** *p*<0.01, *** *p*<0.001 from CT for B and D, *n* = 3 for each experiment.

Since A_1_R is elevated shortly after bacterial inoculation (Figure 1) and is followed by elevation of A_2A_R expression, we wanted to examine whether the induction of A_2A_R by adenosine may be mediated by the A_1_R. Therefore, we treated PMC and PMΦ with 0.1, 1 and 10 µM at 3 hour intervals with A_1_R agonist (CHA) or adenosine in the presence or absence of the A_1_R antagonist (DPCPX, 50 nM). As shown in Figure 3A and B, CHA upregulated mRNA levels of the A_2A_R while treatment with adenosine in the presence of the DPCPX blocked A_2A_R upregulation both in PMΦ and PMC respectively. In contrast, stimulation with CGS, an A_2A_R agonist failed to induce A_2A_R (Figure 3D).

Ligation of adenosine to the A_1_R is mediated through the interaction with members of the G_i_/G_o_ family and inhibits adenylyl cyclase activity. To elucidate the mechanism by which A_1_R induces A_2A_R elevation, we pretreated PMC with PTX, a G_i_ inhibitor (Figure 3C). Pretreatment with PTX blocked the effect of CHA on A_2A_R mRNA levels.

For effective induction of A_2A_R a sequential induction with increasing doses of adenosine or CHA (0.1, 1, 10 µM) were necessary suggesting the involvement of an additional adenosine receptor. CCPA, a specific A_1_R agonist, was less effective than CHA, an A_1_R agonist with lower specificity (Figure 3D). ZM241385, an A_2A_R antagonist, partially blocked the induction of A_2A_R mRNA that was induce by adenosine (Figure 3D) or CHA (data not shown), which suggests that in addition to the requirement of A_1_R stimulation, A_2A_R ligation supports its own induction. Treatment with adenosine in the presence of A_3_R (MRS1220, 100nM) or A_2B_R antagonist (MRS1754, 50nM) did not alter on A_2A_R mRNA levels (data not shown).

### Effect of A_1_R agonist on the expression of A_2A_R and A_1_R *in vivo*

**Figure 4:**
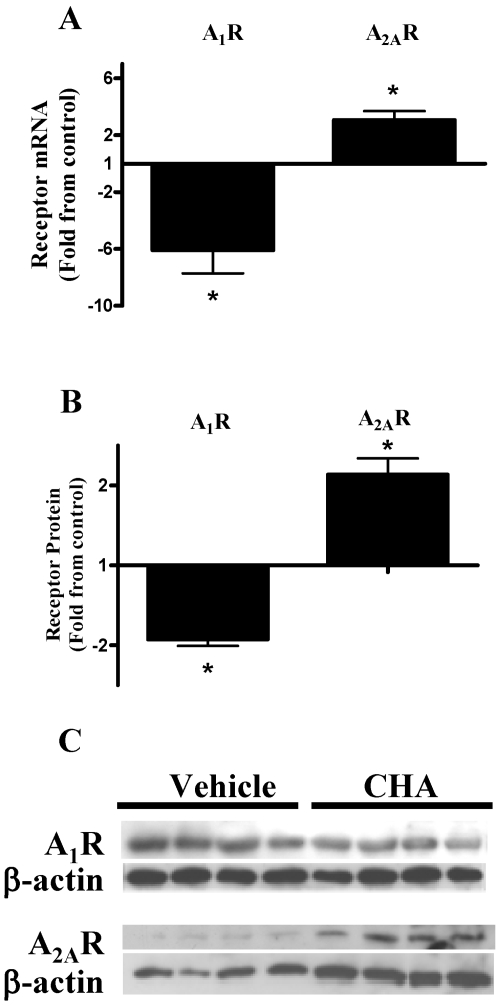
Effect of A_1_R agonist on A_1_R and A_2A_R levels *in vivo.* Mice were administered i.p. with the A_1_R agonist (CHA, 0.1 mg/kg) or with vehicle. PMC were scraped from the peritoneal surface and analyzed for (A) A_2A_R and A_1_R mRNA levels at 4 hours or (B+C) A_2A_R and A_1_R protein levels at 24 hours. (B) Densitometry of protein blot depicted in (C). A_1_R and A_2A_R mRNA levels were normalized to GAPDH and protein levels were normalized to β-actin. Results are presented as fold change from vehicle-treated animals. Data represent three experiments and are expressed as mean±SEM. * *p*<0.05 between conditions per receptor, *n* = 4 for each experiment.

We examine whether the A_1_R agonist also regulates the levels of the A_2A_R *in vivo*. We determined the mRNA and protein levels of the A_2A_R and the A_1_R in mice that were administered an A_1_R agonist (CHA, 0.1 mg/kg). We found that A_2A_R mRNA levels increase ∼3 fold and that A_2A_R protein levels increase ∼2.5 fold, compared to vehicle. In contrast, as shown in Figure 4, both A_1_R mRNA and protein levels decreased in the presence of A_1_R agonist by ∼6 and ∼2 fold, respectively.

### Pretreatment with the A_1_R agonist reduces serum cytokine levels and peritoneal leukocyte recruitment during inflammation

**Figure 5:**
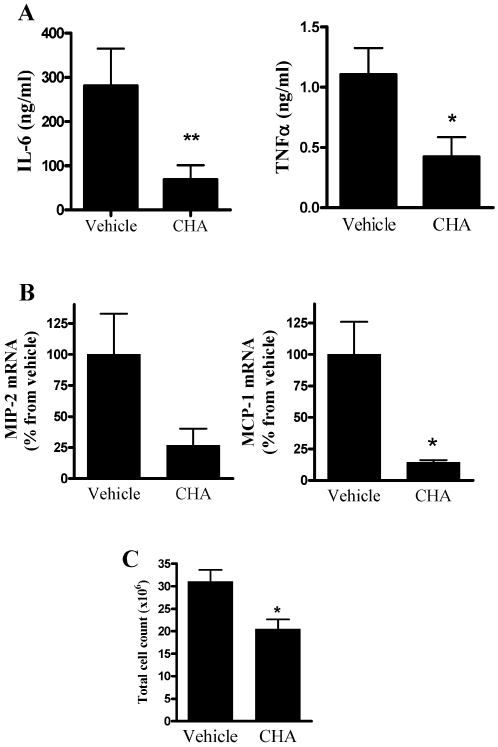
The anti-inflammatory effect of pretreatment with the A_1_R agonist. Mice were treated with the A_1_R agonist (CHA, i.p., 0.1 mg/kg) or vehicle 24 hours prior to bacterial inoculation. (A) Sera levels of IL-6 and TNFα at 12 hours. (B) Chemokine mRNA levels. 12 hours after inoculation PMC were scraped from the peritoneal membrane and total RNA was extracted, analyzed for MCP-1 and MIP-2 mRNA levels and normalized to β-actin. (C) Total cell count at 24 hours after inoculation. Cell exudates were collected from peritoneal lavage fluid. Data represent five experiments and are expressed as mean±SEM for serum cytokine levels and as mean±SEM fold of control for chemokine mRNA levels.* *p*<0.05, ** *p*<0.01, *n* = 5 for each experiment.

Since we showed that A_2A_R levels are upregulated through the activation of A_1_R both *in vitro* and *in vivo*, we wanted to elucidate whether pretreatment of A_1_R agonist before inoculation would upregulate the expression of A_2A_R and lead to advancement of the anti-inflammatory response via A_2A_R. For this, mice were treated with an A_1_R agonist (CHA, 0.1 mg/kg) 24 hours before inoculation of *E. coli*, after which sera were analyzed for IL-6 and TNFα levels. As shown in Figure 5A, we found a significant reduction both in serum IL-6 and TNFα levels 12 hours after inoculation (to 25% and 38% from vehicle, respectively).

Since PMC express an array of chemokines which cause accumulation and activation of leukocytes in tissues, we wanted to examine changes in the levels of CXC chemokines, MCP-1 and MIP-2, following pretreatment with A_1_R agonist. As a result of pretreatment with the A_1_R agonist (CHA 0.1mg/kg), MCP-1 and MIP-2 mRNA level decreased in comparison to vehicle, as determined 12 hours after inoculation (Figure 5B). In accordance with reduced chemokine levels, leukocyte recruitment significantly decreased 24 hours after inoculation to 66% from vehicle, as determined in lavage fluid (Figure 5C).

### A_1_R-agonist preconditioning is blocked by a selective A_1_R antagonist

**Figure 6:**
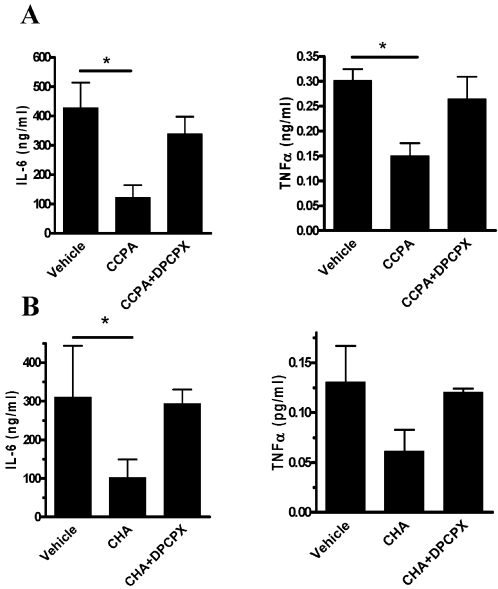
Treatment with A_1_R antagonist blocked the anti-inflammatory effect of A_1_R agonists. 2 hours prior to administration of A_1_R agonist, (A) CCPA (0.1 mg/kg) or (B) CHA (0.02 mg/kg), mice were injected with A_1_R antagonist (DPCPX, 1 mg/kg) or vehicle. After 24 hours, peritonitis was induced by bacterial inoculation. At 12 hours from inoculation, IL-6 and TNFα were analyzed in sera and lavage fluids. Data represent two experiments and are expressed as mean±SEM. * *p*<0.05, between vehicle and CHA or CCPA, *n* = 5 for each experiment.

To ensure that the anti-inflammatory state was mediated by selective activation of the A_1_R, we examined the anti-inflammatory effect of low-dose CHA and an additional specific A_1_R-agonist CCPA, in the presence of a specific A_1_R antagonist (DPCPX). As shown in Figure 6, treatment with either CCPA (A) or CHA (B) significantly reduced serum and lavage IL-6 and TNFα levels. However, pretreatment with an A_1_R antagonist (DPCPX, 1 mg/kg) 2 hours before administration of A_1_R agonist blocked the effect of 0.02 mg/kg CHA, 0.1 mg/kg CHA (data not shown) and 0.1 mg/kg CCPA.

### Modulation of the inflammatory response due to pretreatment with the A_1_R agonist is A_2A_R-dependent

**Figure 7:**
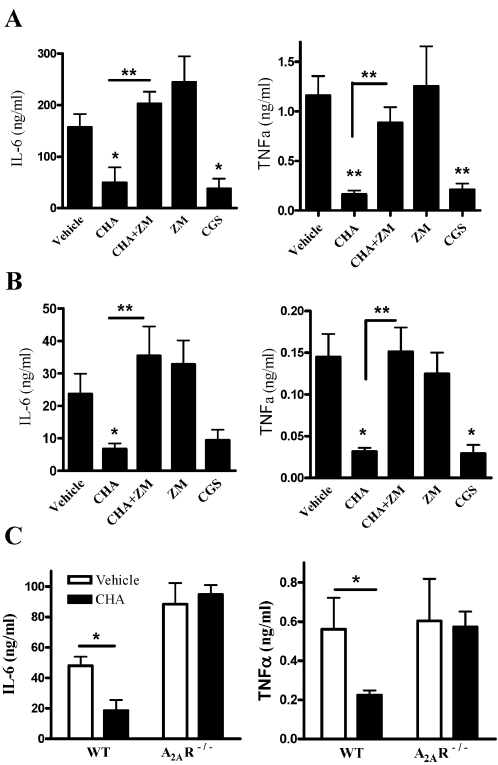
The effect of A_1_R agonist, in A_2A_R^−/−^ and in the presence of A_2A_R antagonist. Mice were administrated with A_1_R agonist (CHA, 0.1 mg/kg) or vehicle 24 prior to bacterial inoculation. 30 min before inoculation the A_2A_R antagonist (ZM241385, 1 mg/kg) or the A_2A_R agonist (CGS21680, 1 mg/kg) were administered to the same animals or to untreated animals. (A) sera IL-6 and TNFα (12 hours) and (B) lavage fluids IL-6 and TNFα (12 hours). (C) A_2A_R^−^^/−^ mice or their WT littermates were treated with the A_1_R agonist (CHA, 0.1 mg/kg) i.p. or vehicle 24 hours prior to bacterial inoculation. 12 hours following inoculation sera were collected and analyzed for IL-6 and TNFα levels. Data are representative of three individual experiments and are expressed as mean±SEM. * *p*<0.05, ** *p*<0.01 between vehicle and CHA or CGS21680 and between CHA with or without ZM241385, *n* = 5 for each experiment.

To prove that the modulation in the inflammatory response (Figure 5) is mediated by A_2A_R, we treated animals with an A_2A_R antagonist (30 min before inoculation, ZM241385, 1 mg/kg). As shown in figure 7, blockade of the A_2A_R caused an increase in serum and lavage IL-6 and TNFα levels to similar levels found in infected mice administrated with vehicle alone. As expected, administration of A_2A_R agonist (30 minutes before inoculation, CGS21680, 1 mg/kg) reduced IL-6 and TNFα levels in serum and lavage fluids to levels comparable to those found in CHA-treated animals. In concordance, pretreatment of A_2A_R^−/−^ mice with A_1_R agonist resulted in unchanged serum IL-6 and TNFα levels (Figure 7C), as well as chemokine mRNA levels in PMC (data not shown). However, in WT mice there was a significant reduction both in cytokine levels and mRNA chemokine levels (data not shown). These data suggest that the modulation of the inflammatory response caused by pretreatment with A_1_R agonist is, indeed, mediated by A_2A_R.

## Discussion

The study presented here demonstrates a novel mechanism of adenosine receptor subtype autoregulation. Since adenosine action is mediated through at least four different receptors, each of which exhibits a unique affinity and opposing signaling pathways, the regulation of subtypes expression is critical for determining the outcome of adenosine activity [5]. Others and we have shown that adenosine receptors are regulated by various inflammatory mediators and multiple endogenous factors [24]. For example, we found that A_2A_R mRNA and protein levels are upregulated in human PMC following treatment with IL-1β and TNFα, while treatment with IFNγ strongly decrease A_2A_R expression both alone and in combination with IL-1β and TNFα [19]. In the same study, we show that following inoculation, adenosine receptor levels on PMCs are sequentially upregulated and that adenosine is induced following inoculation and reaches peak levels at 24 hours [19]. The A_1_R is induced during the first phase of leukocyte recruitment and the A_2A_R is induced later, at the resolution phase of peritonitis [19]. In the present study, we obtained the same pattern of adenosine receptor expression on peritoneal leukocytes. These results suggest that both mesothelial cells and the recruited leukocytes are highly synchronized in their response to adenosine. Furthermore, this sequential elevation of the A_1_R and the A_2A_R on PMC and leukocytes suggests that adenosine may regulates its receptors. Both our *in vitro* and *in vivo* data in the current study support this suggestion; we found that adenosine significantly upregulates A_2A_R expression levels in isolated PMC in a dose dependent manner.

Of all adenosine receptor subtypes, A_1_R exhibits the highest affinity for adenosine (K_i_ = 10 nM) [34], implying that A_1_R is activated at the low levels of adenosine produced during the initiation of inflammation. This early activation of A_1_R receptor may enable the induction of A_2A_R. The A_1_R agonist, CHA, significantly induced the expression of A_2A_R, while treatment with the A_1_R antagonist, DPCPX, or with PTX, a G_i_ inhibitor, blocked A_2A_R induction by adenosine, indicating that A_1_R ligation is necessary for the induction of A_2A_R. Treatment with CGS21680, an A_2A_R agonist, did not induce the expression of the A_2A_R. However, treatment with the A_2A_R antagonist in the presence of adenosine partially blocked A_2A_R induction. Therefore, one can conclude that A_2A_R ligation by elevated levels of adenosine is required to support the initial signal of A_1_R.

According to our *in vitro* data, mice treated with CHA exhibited a significant 2-3 fold increase in A_2A_R mRNA and protein levels as determined, in PMCs compared to untreated animals. Interestingly, mRNA and protein A_1_R levels were significantly down-regulated by these same treatments in PMCs (6- and 2-fold decrease, respectively), suggesting that A_1_R receptor may be responsible for the “switching” between the two receptor subtypes during inflammation. In Support of our findings, Schnurr et al. showed that in immature plasmacytoid dendritic cells (PDCs) adenosine activates A_1_R, which induces chemotaxis; however, in mature PDCs, A_1_R is replaced by the A_2A_R, which inhibits cytokine production [9].

In order to understand the physiological role of the exchange between the two receptors, we examined whether ligation of the A_1_R will trigger the induction of the A_2A_R and lead to an advancement of the resolution phase of the inflammatory process. We found that preconditioning with an A_1_R agonist significantly reduces the inflammatory response to bacterial challenge. CHA or CCPA administration at 24 hours before inoculation significantly reduced sera and peritoneal levels of the pro-inflammatory cytokines TNFα and IL-6, and reduced mRNA levels of chemokines on PMC as well as leukocyte recruitment to the peritoneum. The anti-inflammatory effect induced by pre-treatment (24 hours) with A_1_R agonist was also achieved by a specific A_2A_R agonist (CGS21680) administered to animals 30 minutes before bacterial inoculation. Pre-treatment with CHA or CCPA had no anti-inflammatory effect in animals that were administered with the A_1_R antagonist, DPCPX 2 hours before agonists or A_2A_R antagonist, ZM241385 30 minutes before inoculation or when A_2A_R^−\−^ animals were examined. The marked blocking effect of ZM241385 and the lack of effect of CHA in A_2A_R knockout animals clearly indicate that the anti-inflammatory effects of the A_1_R agonist are mediated by the A_2A_R.

**Figure 8:**
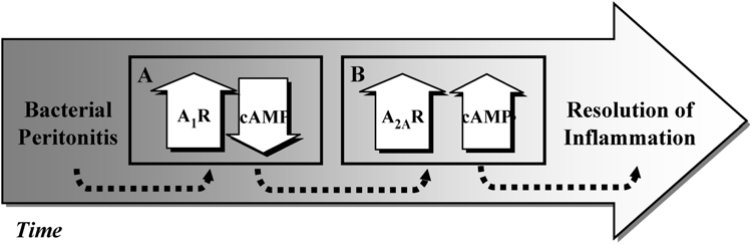
Effect of adenosine receptor subtype autoregulation on the inflammatory process. (A) Early expression of A_1_R after bacterial inoculation decreases cAMP levels, enhances production of local pro-inflammatory cytokines and promotes leukocyte migration. (B) In a later phase of peritonitis A_2A_R expression increase by A_1_R which leads to increase in cAMP levels. High cAMP markedly decreases local pro-inflammatory cytokines and leukocyte recruitment, hence restraining inflammatory flames.

Elevation of cAMP usually down-regulates the inflammatory response [5]. Since A_1_R is a G_i_ coupled receptor that suppresses the induction cAMP, it is not surprising that this receptor had no direct anti-inflammatory effect. High expression of A_1_R implies that immediately after inoculation, decreased cAMP levels give rise to local pro-inflammatory cytokines and leukocyte migration, hence allowing an adequate and effective immune response to the invading microorganisms. In contrast, the increase in A_2A_R at late phases of peritonitis is probably associated with elevated cAMP levels, which markedly decrease local pro-inflammatory cytokine levels and leukocyte recruitment, hence restraining inflammatory flames (Figure 8).

In summary, our study sheds light on the sequential autoregulation of adenosine receptor subtypes. The mechanism we have describes may directly participate in the propagation of the compensatory anti-inflammatory response syndrome (CARS), which follows systemic inflammation in trauma patients. Whether patients with CARS exhibit elevated adenosine levels pursuing traumatic insult should be explored. These findings may also have future implications for clinical treatments by combining pre-treatment with an A_1_R agonist and subsequent A_2A_R agonist to enhance the anti-inflammatory effect, or to promote anti-inflammation by endogenous adenosine at the site of inflammation. As such, preconditioning with an A_1_R-agonist could be used in preparation of tissue for transplantation or to induce an anti-inflammatory and immunosuppressive state in patients before invasive surgery and organ transplantation.
